# Investigation of the biomechanical effect of variable stiffness shoe on external knee adduction moment in various dynamic exercises

**DOI:** 10.1186/1757-1146-6-39

**Published:** 2013-09-17

**Authors:** Jee Chin Teoh, Jin Huat Low, Ying Bena Lim, Victor Phyau-Wui Shim, Jaeyoung Park, Seung-Bum Park, Sang Jun Park, Taeyong Lee

**Affiliations:** 1 Department of Bioengineering, Faculty of Engineering, National University of Singapore, Block E3A #07-15, 7 Engineering Drive 1, Singapore 117574, Singapore; 2 Department of Mechanical Engineering, Faculty of Engineering, National University of Singapore, Singapore, Singapore; 3 Department of leisure sports, Dongeui University, Busan, Korea; 4 Footwear Biomechanics Team, Footwear Industrial Promotion Center, Busan, Korea; 5 Lecaf Footwear Planning Department, Hwa Seung Corp. Ltd., Seoul, Korea

**Keywords:** Knee osteoarthritis (OA), Variable stiffness shoe (VSS), External knee adduction moment (EKAM)

## Abstract

**Background:**

The growing ageing population and high prevalence of knee osteoarthritis (OA) in athletes across nations have created a strong demand for improved non-invasive therapeutic alternatives for knee OA. The aim of this study is to investigate the effect of the variable stiffness shoe (VSS), a new non-invasive therapeutic approach, on external knee adduction moment (EKAM) in various dynamic exercises. EKAM is believed to have positive correlation with the progression and development of knee OA.

**Methods:**

Thirty young participants (16 male and 14 female; age 22.6 ± 1.9 years) from National University of Singapore were enrolled in this study. The tested activities were walking, running, drop-landing, and lateral hopping. All the dynamic exercises were recorded simultaneously by the 8-camera VICON Motion Systems (Oxford Metric, UK) with a sampling rate of 100 Hz.

**Results:**

The results showed that the EKAM was reduced in all the dynamic exercises with the use of VSS. The VSS produced significant reductions in the peak EKAM during walking (4.97%, *p* = 0.039), running (11.15%, *p* = 0.011), drop-landing (11.18%, *p* = 0.038) and lateral hopping (17.34%, *p* = 0.023) as compared to the control shoe.

**Conclusions:**

The reduction of EKAM with the use of VSS in various dynamic exercises demonstrates its potential in delaying the onset and the progression of knee OA in early stage of knee OA patients.

## Background

Osteoarthritis (OA) is the most prevalent joint disorder in the world [1], resulting from the biochemical breakdown of articular cartilage in the synovial joints. At present, 33.6% of the U.S. population aged above 65 are affected by this condition [2]. Research also shows that athletes are more prone to the early onset of knee OA with 3.3% prevalence of OA in elite athletes compared to 1.4% in non athletes [3] due to periodic mechanical impact on knee during sports activities [4-7]. Vigorous sports such as football, rugby, and hockey are found to expose the players to higher risk of developing OA [7,8].

Out of the many joints in the human body, knee OA is the most common type of osteoarthritis [2]. 25% of knee OA population have difficulty in walking 1/4 mile and climbing the stairs while 15% have to use assistive device such as a cane for walking [9]. The profound impact of knee OA on quality of life and the serious implication of total joint replacement have led to great research interest in designing new preventive approaches.

The majority of knee OA is diagnosed in the medial condyle of the knee [10]. During walking, the forces passing through the medial compartment can be 2.5 times greater than that through the lateral compartment [11,12]. Tetsworth and Paley [13] postulated a 4-6% increase in varus alignment will further increase the medial loading across the knee joint by 20%. Malalignment of the knee, by altering the load distribution, can hence be deemed as a risk factor of knee OA [13].

The external knee adduction moment (EKAM) is the product of ground reaction force (GRF) and the moment arm with respect to knee joint center. It has been demonstrated to be a valid indicator for medial compartment loading [14-17]. An increase in EKAM causes adduction at the tibiofemoral joint and eventually elevates compressive load at the medial compartment of the knee joint. This rise of joint forces is undesirable as it results in a negative deleterious effect on knee cartilage and contributes to development and progression of OA [12,18-24].

It was reported in the study by Sharma et al. [24] that the widely used OA pathogenesis could be categorized into two, i.e. (1) Increased regional load across the articulating joint and (2) altered material properties of articular cartilage which affect its ability to withstand compressive load. Due to the strong association of EKAM and medial loading, as well as the causative link between medial loading and development of knee OA, many believe that greater EKAM values indicated higher risk in knee OA [12,25-28]. In line with this, EKAM can be a predictor of knee OA development [28]. Hence, it is important to study how EKAM changes under different therapeutic approaches in order to examine their effects in reducing risk of developing knee OA.

On the other hand, Vanwanseele et al. [29] demonstrated that no associations were found between the severity of OA and the EKAM although significantly higher EKAM and knee adduction angular impulse were observed in the subgroup of medial OA patients. Furthermore, a case-study using instrumented knee prosthesis showed that a reduction in first peak EKAM does not guarantee a reduction in medial contact load [29] and Bennell et al. [30] also showed that medial knee load was not affected by EKAM. Therefore, changes in EKAM may not accurately reflect changes in knee OA progression. Direct measurements of changes in medial contact force seems to be a more appropriate way to determine the effectiveness of therapeutic approaches on knee OA progression, nonetheless, the in vivo knee contact forces cannot be easily measured. Hence, EKAM is still a good predictor of medial knee OA progression [28] to examine the effect of VSS. Erhart et al. [31] indicated that EKAM peak was significantly correlated with decreases of medial peak force in a participant with a total knee replacement.

Due to the discomfort and inconvenience faced by people with OA, pain alleviation and functional improvement have become the primary goals of OA therapy. Many approaches have been taken to reduce EKAM in attempts to relieve pain and prevent the development or progression of knee OA. These methods range from non-invasive techniques, such as gait modification, laterally wedged insoles [32-34] and variable stiffness shoe [31,35], to an invasive approach such as high tibial osteotomy [36-38]. The effects of these approaches are presented in Table 1. As therapeutic footwear is a non-invasive treatment and can be easily utilized, it is deemed the first-line approach for knee OA disease [39] as well as to prevent the onset of this disease.

**Table 1:** A summary of the biomechanical effect of interventions presented in the literature

**Intervention**	**Author**	**Subject**	**Reductions in 1**^**st **^**Peak of EKAM**
High Tibial Osteotomy (surgical intervention)	Bhatnagar *et. al*[36]	30 HTO patients	58% (No significant difference between 6 and 12 months post-HTO)
	Birmingham *et. al*[37]	126 patients with knee OA	46%
	Weidenhielm *et. al*[38]	9 patients after high tibial valgus osteotomy	64%
Laterally Wedged Insoles (Footwear intervention)	Shimada *et. al*[32]	23 patients with medial knee OA	4.4%
	Kerrigan *et. al*[33]	15 patients with clinical and radiographic OA	6% (in 5° wedge)
			8% (in 10° wedge)
	Crenshaw *et. al*[34]	15 normal healthy subjects	7%
Variable Stiffness Shoe (Footwear intervention)	Jenkyn *et. al*[35]	32 subjects with medial compartment knee OA	6.6%
	Erhart *et. al*[31]	1 subject with total knee replacement	13.3%

Some participants have voiced their discomfort after using the laterally-wedged insoles [33]. Alternatively, the variable stiffness shoe (VSS), with a stiffer lateral midsole, can potentially be a better approach to reduce EKAM without causing additional discomfort on shoe wearers. Current research has shown the efficacy of VSS in reducing the EKAM and decreasing the knee pain of patients wearing VSS during walking [31,35,40,41]. However, the biomechanical effects of VSS in other dynamic exercises such as running, drop-landing and lateral hopping has yet to be investigated. In fact, it has been reported that men aged between 20–49 who ran more than 20 miles per week had 2.5 times higher risk in developing OA, as compared to sedentary men [42]. Studies showed that long term vigorous activities increase the incidence of OA [42-50]. Nonetheless, there are also reports that indicated no significant increase in risk of OA from physical exercises [51-58]. The relationship between physical activities and OA development is unclear.

Despite the contrary, repetitive movement in exercise is still thought to have potentially adverse effect on knee cartilage due to increased joint forces. Hence it is necessary to investigate the effect of VSS in reducing EKAM during various dynamic exercises. In addition, given the fact that several reports have demonstrated higher knee OA risk in athletes [4-7], biomechanical effects of VSS in dynamic exercises are not well established. It was hypothesized that the EKAM would be lowered during locomotion with the use of VSS.

## Methods

### Participants

**Figure 1:**
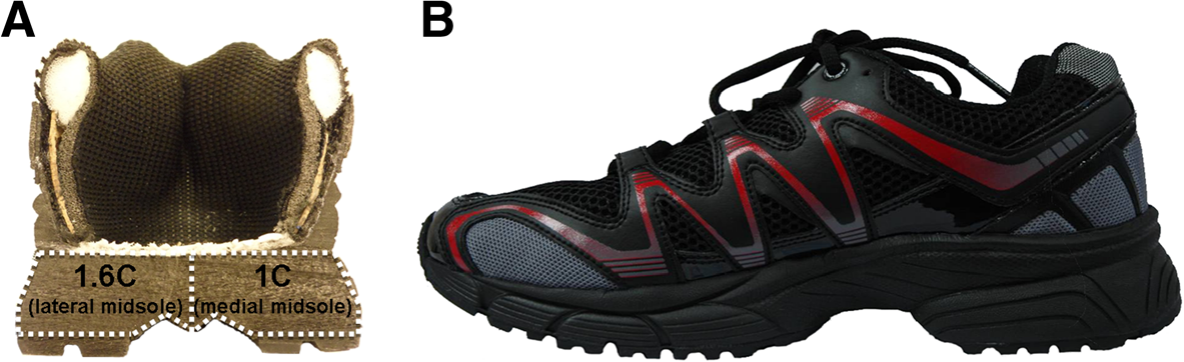
**Variable stiffness shoe with the lateral midsole (1.6C) that is 1.6 times stiffer than the medial midsole (1C). (A)** section view, **(B)** side view.

Thirty young participants (16 male and 14 female; age 22.6 ± 1.9years) from National University of Singapore (NUS) were enrolled in this study. Participants with any history of lower extremity musculoskeletal pathology, serious injury to lower extremity and back, or OA in lower extremity joints were excluded. Participant characteristics such as weight and body mass index (BMI) are presented in Table 2. The study was approved by the NUS Institutional Review Board (NUS-IRB) (reference code: 11-264). Participants were required to perform several trials of different dynamic activities using variable stiffness shoe (VSS) (Figure 1) with a medial: lateral stiffness ratio of 1:1.6. This ratio was selected based on the finite element modeling of an anatomically detailed 3D FE model of the male foot and ankle [59,60] incorporated with a midsole support made of compression molded ethylene vinyl acetate. The foot model was constructed based on CT images and was solved using ABAQUS (SIMULIA). It was found that the lateral ankle and medial knee pressure were inversely correlated. The ratio was optimized between these two variables. To examine the effects of the VSS objectively, participants were also asked to perform trials with a constant-stiffness (1:1 stiffness ratio) control shoe (New Balance, Boston MA, USA). In addition, participants were blinded to the shoe type and the order of shoe testing was randomized. All the motion analyses were conducted at the NUS bioengineering gait laboratory.

**Table 2:** Participant characteristics

	**Participants (n = 30)**
Gender	16 M / 14 F
Mean age (SD)	22.6 (1.9) years
Body weight (SD)	57.2 (7.9) kg
Body Mass Index (SD)	20.68 (2.23) kg/m^2^

### Gait analysis protocol

All the dynamic exercises were recorded simultaneously by the 8-camera VICON Motion Systems (Oxford Metric, UK). Two side-by-side force plates (AMTI, Massachusetts, US) embedded in a 12m walkway were used to record the magnitude and direction of the ground reaction force (GRF) present during the motion. Both kinematics and kinetics data were sampled at a sampling rate of 100 Hz. A total of sixteen markers were placed on the lower extremities of each participant. Two markers were affixed to each shoe at the second metatarsal head and heel region respectively. Other markers were placed on the ankle, tibia, knee, thigh, anterior superior iliac spine and posterior superior iliac spine. All the markers attached on the skin remained on the participant throughout the entire session in order to minimize the variations in angles and external moments caused by different anatomical reference frame. The kinetic data such as knee joint moments and GRF were retrieved and processed using spreadsheet software.

Participants were required to perform four dynamic exercises listed below in two shod conditions, the VSS and the constant stiffness shoe.

**Figure 2:**
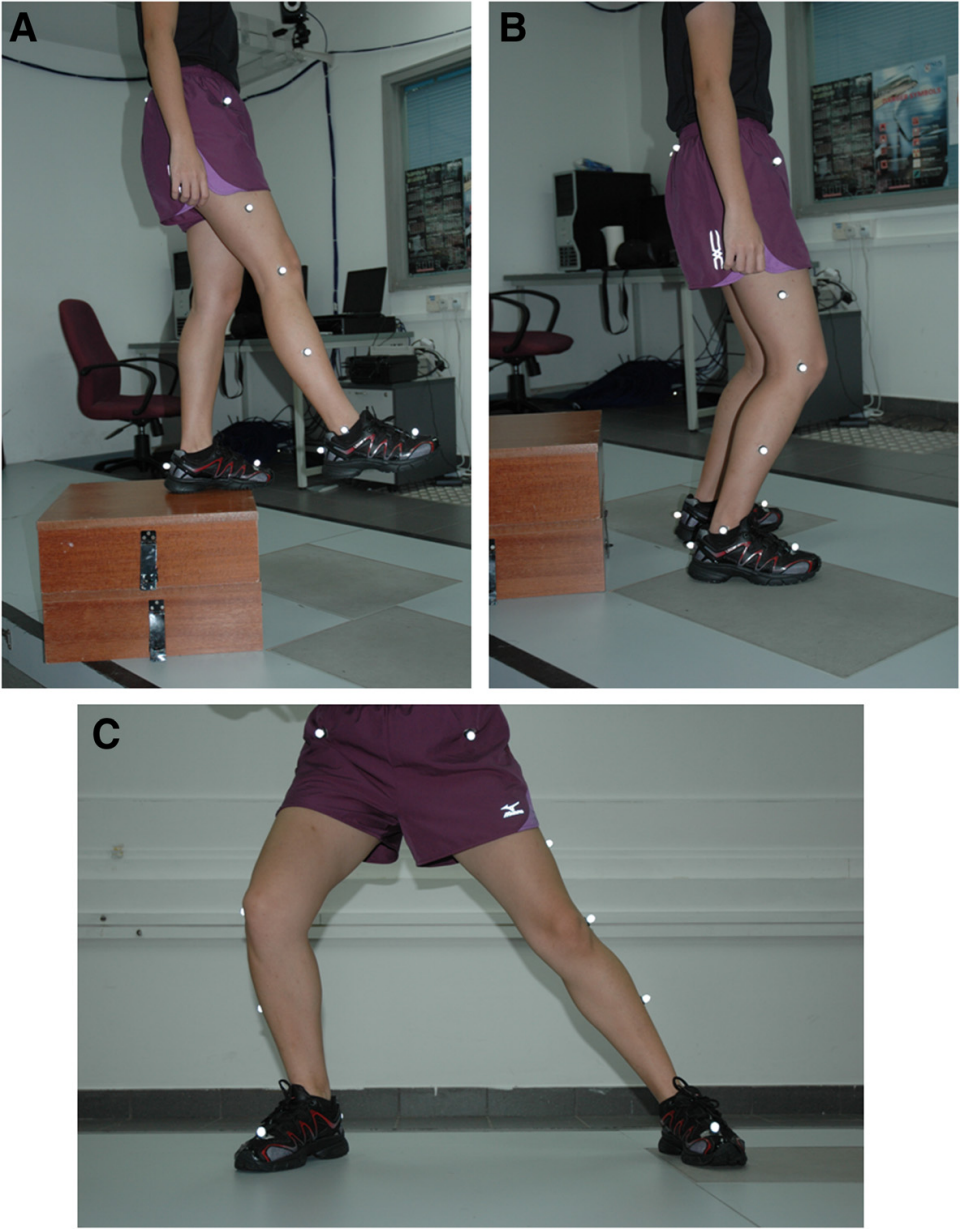
The different dynamic exercises tested in this experiment (A, B) drop-landing, (C) lateral hopping.

(1) Walking: Participants were instructed to walk at their natural walking speeds with each foot striking one of the force plates respectively.

(2) Running: Participants were tasked to run at their self-selected speeds with their left foot achieving contact with the force plate.

(3) Drop-landing (Figure 2A, 2B): Participants were asked to perform the drop-landing task at a height of 30cm, beginning from a standing position on a platform. They were subsequently instructed to step off the platform (leading with their dominant leg) and land with each foot on each force plate simultaneously.

(4) Lateral hopping (Figure 2C): Participants were tasked to carry out 3–5 lateral hops before striking the center of the force plate positioned 1.5 m away from the starting point.

All the data collection was preceded by adequate familiarization. Trials were omitted if participants were found to have obvious gait adjustments secondary to visual targeting of the force plates. Three successful trials from each participant were recorded for data analysis.

In addition, the 7-point perception scale questionnaire was also conducted to evaluate the comfort level of the VSS. Three aspects of shoes were rated, i.e. overall comfort, cushioning and stability. Participants were asked to grade the shoes after each activity, 1 being extremely dissatisfied and 7 being extremely satisfied.

### Data analysis

All kinetic and kinematic data were averaged for each participant across three trials. GRF data were normalized to body weight (%BW) and the EKAM was normalized to body weight and height (%BW x height) to eliminate intergroup difference. Maximum GRF and EKAM values at the characteristic peaks during each dynamic exercise were obtained from the average curves. The average curves were graphed against the percentage stance in the walking case and graphed against time in other three exercises.

Paired t-tests were used to identify significant differences between the control shoe and the VSS for each dynamic activity. Percentage differences were normalized by the control shoe. Statistical significance was considered as *p* < 0.05.

## Results

**Figure 3:**
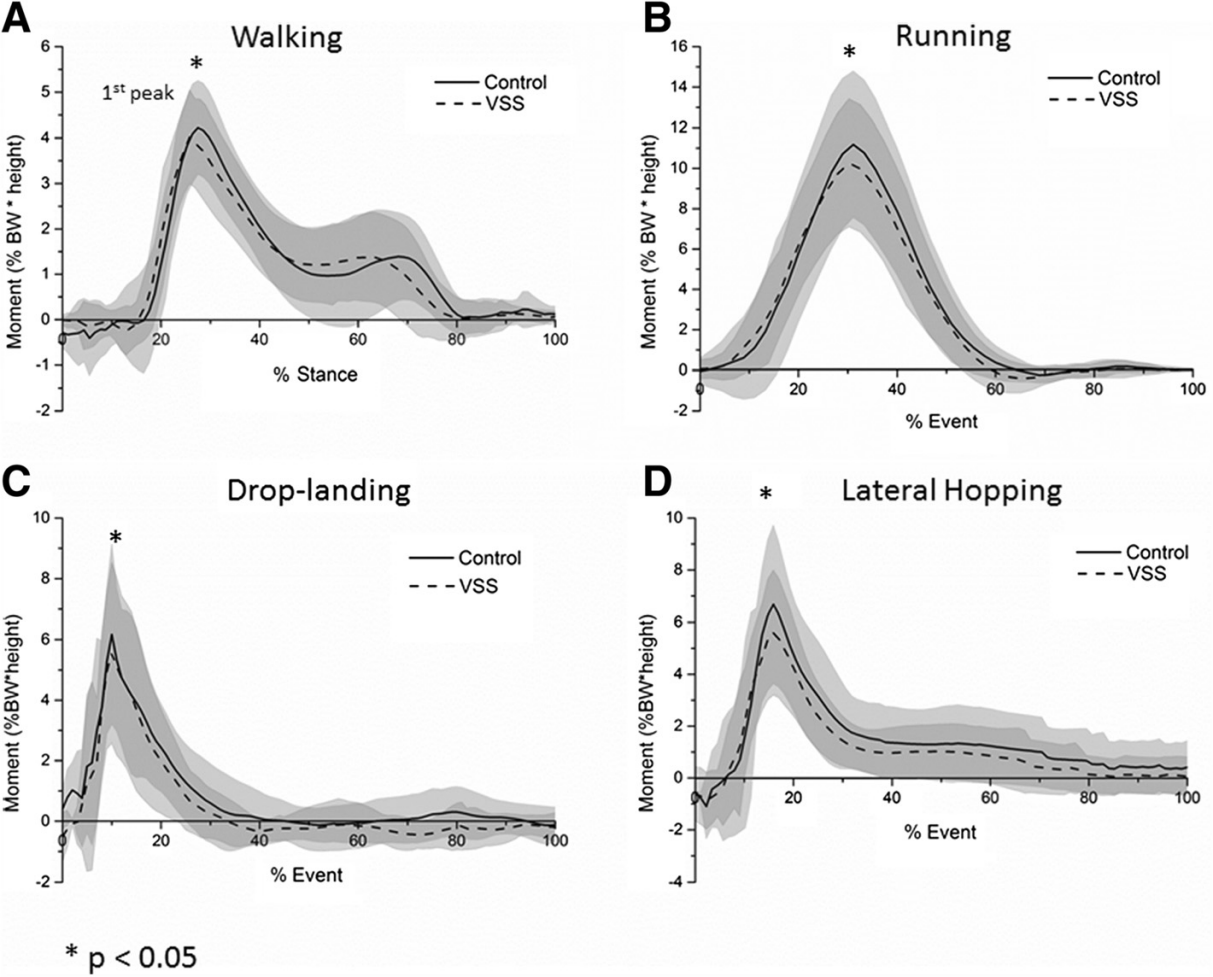
**The effect of shoe intervention on external knee adduction moment in (A) walking, (B) running, (C) drop-landing and (D) lateral hopping.** *p < 0.05.

In this study, there were no significant differences in walking and running speed between the shod conditions. Walking speed for the control condition was 1.13 ± 0.17 m/s, and for the VSS condition was 1.22 ± 0.11 m/s [*p* = 0.104]. Running speed for the control condition was 2.45 ± 0.32 m/s, and for the VSS condition was 2.4 ± 0.33 m/s [*p* = 0.149]. The EKAM profiles in two shod conditions during the four tested activities are as presented in Figure 3. The VSS produced a significant reduction in the EKAM (4.97%, *p* = 0.039) at the first peak during walking as compared to the control shoe. There was also a decrease in the EKAM at second peak during walking with VSS but the difference was not statistically significant. Results in Table 3 also reveal that the VSS produced significant reductions in the peak EKAM during running (11.15%, *p* = 0.011), drop-landing (11.18%, *p* = 0.038) and lateral hopping (17.34%, *p* = 0.023) as compared to the control shoe.

**Table 3:** Group-mean (standard deviation) values of GRF during running

**Variable name (%BW)**	**Control shoe**	**VSS**	**% difference**	***P*-value**
Max Medial GRF	17.45 (4.66)	15.46 (4.16)	−11.42	0.013^*^
Posterior GRF	−19.33 (5.73)	−21.75 (8.5)	12.52	0.147
Anterior GRF	29.32 (4.68)	32.91 (5.4)	12.21	0.010^*^
Max Vertical GRF	220.74 (23.52)	217.14 (21.82)	−1.63	0.131

*Significant at *p* < 0.05.

The kinetic data (GRF) obtained from the running task is as tabulated in Table 4. The VSS produced significant reductions (11.42%, *p* = 0.013) in the maximum medial GRF. However, there was a significant increase in anterior GRF. There was also an increase in the posterior GRF but the difference was not statistically significant. Findings from the 7-point perception scale questionnaire show that the comfort of the participants was not compromised with the use of the VSS as compared to the control shoe.

**Table 4:** Group-mean (standard deviation) values of EKAM in two different shoe conditions

**Activities**	Variable name (%BW*ht)	**Control shoe**	**VSS**	**% difference**	***P*-value**
Walking	EKAM (1^st^ peak)	4.23 (1.01)	4.02 (1.03)	−4.97	0.039^*^
	EKAM (2^nd^ peak)	2.00 (0.67)	1.97 (0.78)	−1.64	0.422
Running	Max EKAM	11.56 (3.32)	10.27 (3.16)	−11.15	0.011^*^
Drop-landing	Max EKAM	6.2 (2.92)	5.5 (3.08)	−11.18	0.038^*^
Lateral hop	Max EKAM	6.75 (3.14)	5.58 (2.35)	−17.34	0.023^*^

*Significant at *p* < 0.05.

## Discussion

The objective of this study was to investigate the changes in EKAM with the use of VSS during various dynamic exercises. In spite of the rising incidence of knee OA among athletes worldwide, only the reduction in EKAM throughout stance during walking has been well documented in both healthy participants and participants with different stages of knee OA. To our knowledge this is the first study to report on the reduction in EKAM during other dynamic activities such as running, drop-landing and lateral hopping.

The 5% reduction in peak EKAM during walking in this study is consistent with, or slightly smaller than, that of participants with medial knee OA (KL grades 1–2) studied by Jenkyn et al., of 6.6% [35] and Erhart et al., of 7% [41]. However, Erhart et al. [31] found reductions in EKAM, ranging from 13% at the 1^st^ peak to 22% at the 2^nd^ peak, during walking with the VSS. The significantly greater reduction in EKAM observed in that study may be due to either the use of a single participant with a total knee replacement or the gait adaptations undergone by the patient with the implanted knee. Furthermore, this significant reduction in the peak EKAM contradicts the findings of a recent published study by Erhart et al. [41] in which no significant difference in peak EKAM was found between control and intervention group with severe medial knee OA (KL grades 3–4). An explanation for these differences may be the BMI and age of participants, the walking speed, or the severity of medial knee OA. It is also possible that the VSS is less likely to affect joint moments with a greater varus knee alignment which is usually found in patients with severe knee OA. This explanation, together with the findings reported by Erhart et al. [40], is consistent with that from a study conducted with the lateral-wedged insole whereby no significant reduction in EKAM was also observed in the participants with severe knee OA during walking [32]. Moreover, the significant reduction in the peak EKAM is in line with the findings reported by Bennell et al. [61]. However, EKAM was also found to be significantly higher in variable stiffness shoe condition as compared to barefoot walking. Since walking barefooted may be impractical under certain circumstances, VSS that resulted in a decrease in EKAM as compared to normal shoe can still be an optimal footwear option for knee OA patients [39].

The double hump pattern exhibited by the EKAM during walking was not exhibited by the EKAM during running. Instead, there was only a single peak found in the graph of EKAM during running. This may due to the participants who are forefoot strike runners, resulting in the absence of impact peak in the GRF as compared to the typical rear foot strike runners.

The peak EKAM was also significantly reduced in other dynamic exercises (11.18% in drop-landing and 17.34% in lateral hopping). Whilst no direct numerical comparison could be made because this is the first study to report these data, other investigators of VSS have proposed mechanisms that can potentially explain the reduction in peak EKAM during these activities. Boyer et al. reported that the greater ankle eversion angle, the less pelvic obliquity and that hip adduction angle results in lower EKAM during walking by changing the posture of the leg in frontal plane [40]. Such a mechanism is likely to be present during the period of landing and lateral hopping when participants attempt to achieve stability.

The potential mechanism responsible for the decrease in EKAM observed with VSS during walking was presented by Jenkyn et al. [35]. They proposed that the reduction in EKAM is due to the reduction in medial GRF, lateral movement of COM due to dynamic adaptation to the differential stiffness from lateral to medial midsole and the reduction in lever arm caused by the eversion moment of the foot that induces a valgus thrust at the knee. We speculated that the same mechanism is applied on other dynamic exercises apart from walking. It is because the stiffer lateral midsole of the VSS will cause eversion at the ankle joint which eventually leads to the reduction in lever arm. However, the exact mechanism by which the EKAM is reduced was not conclusively demonstrated and future study is needed to find out the exact mechanism.

Furthermore, the VSS also caused an increase in anterior GRF by 12.21% during running. This observation suggests that the participants altered their running gait with the use of VSS such that forward acceleration is enhanced. Such an alteration in running gait is crucial for improving the performance of athletes. Despite the promising results obtained, this study needs to be viewed in light of a few limitations. The main limitation is that participants had a limited adaption period to familiarize themselves with the usage of the VSS before test. This limitation could compromise the accuracy of the findings due to the indirect gait pattern changes caused by unfamiliar footwear. However, it has been demonstrated by Erhart et al. that the reduction in the external knee adduction moment due to the VSS is immediate and sustained for at least a period of 1 year while wearing the shoe [41]. Furthermore, only young healthy participants were involved in the study, the results presented may not be representative of elderly population who are more prone to knee OA [62]. However, recruitment of senior participants may impose another limitation to the study, e.g. gait instability, which may reduce the result accuracy. Another source of error may come from the assessment technique applied. Motion capture using plug in gait model was preferred despite of the several potential errors associated, such as anatomical marker misplacement and soft tissue artifact. To reduce these undesirable errors, marker placements were done by the same trained personnel. At least 5 trials were conducted on each activity to examine experiment repeatability. The best 3 trials were then averaged for comparison purpose.

Acknowledging the limitations of this study, there remains a vast potential in the use of VSS as a means of treatment for early stages of knee OA. Future studies could be directed at a larger participant pool to achieve power to draw conclusions on the differences in EKAM between the intervention and control groups as well as the long-term efficacy of VSS in athletes and osteoarthritic participants. In addition, people with medial knee OA have been reported to have a more pronated foot type compared to normal people [63]. Thus, the VSS may have different effects on this population. Apart from the EKAM, future efforts can also be considered to study other variables in this custom-made shoe design such as stress distribution at hip and ankle joint, plantar soft tissue stiffness after prolonged usage of this intervention [64] as well as the vertical and shear forces acting on the foot [65] which are important factors contributing to foot ulceration.

## Conclusions

The findings support the hypothesis that the VSS reduces EKAM during dynamic exercises. Furthermore, the results also demonstrate that the change in EKAM may due to the reduction in medial GRF. As the reduction in EKAM may help to delay the onset and the progression of knee osteoarthritis, it can thus be speculated that the VSS might have the potential to serve as a therapeutic intervention for early stage of medial compartment knee OA, thereby delaying the need for invasive surgery.

Interestingly, the VSS also caused an increase in anterior GRF during running. This effect is beneficial to the forward propulsion of athletes and can potentially enhance the performance in various sport activities. However, the exact mechanism behind this remains unknown and further investigation is required to evaluate the effect of VSS in sports performance.

## Consent

Written informed consent was obtained from the patient for the publication of this report and any accompanying images.

## Competing interests

The authors declare that they have no competing interests.

## Authors’ contributions

JC, JH and TY designed the study. JC, JH and BL conducted the experiment and the statistical analysis. JC drafted the manuscript with assistance from JH, BL, VPWS, SB, SJ and TY. All authors reviewed and approved the final manuscript.
